# The pursuit of further miniaturization of screen printed micro paper-based analytical devices utilizing controlled penetration towards optimized channel patterning

**DOI:** 10.1038/s41598-021-01048-1

**Published:** 2021-11-02

**Authors:** Hsiu-Yang Tseng, Jose H. Lizama, Yi-Wei Shen, Chiu-Jen Chen

**Affiliations:** Department of Mechanical Engineering, National Taiwan University of Science and Technology, Taipei, 106 Taiwan

**Keywords:** Biomedical engineering, Mechanical engineering

## Abstract

One of the main objectives of microfluidic paper-based analytical devices is to present solutions particularly, for applications in low-resource settings. Therefore, screen-printing appears to be an attractive fabrication technique in the field, due to its overall simplicity, affordability, and high-scalability potential. Conversely, the minimum feature size attained using screen-printing is still rather low, especially compared to other fabrication methods, mainly attributed to the over-penetration of hydrophobic agents, underneath defined patterns on masks, into the fiber matrix of paper substrates. In this work, we propose the use of the over-penetration to our advantage, whereby an appropriate combination of hydrophobic agent temperature and substrate thickness, allows for the proper control of channel patterning, rendering considerably higher resolutions than prior arts. The implementation of Xuan paper and nail oil as novel substrate and hydrophobic agent, respectively, is proposed in this work. Under optimum conditions of temperature and substrate thickness, the resolution of the screen-printing method was pushed up to 97.83 ± 16.34 μm of channel width with acceptable repeatability. It was also found that a trade-off exists between achieving considerably high channel resolutions and maintaining high levels of repeatability of the process. Lastly, miniaturized microfluidic channels were successfully patterned on pH strips for colorimetric pH measurement, demonstrating its advantage on negligible sample-volume consumption in nano-liter range during chemical measurement and minimal interference on manipulation of precious samples, which for the first time, is realized on screen-printed microfluidic paper-based analytical devices.

## Introduction

Due to their affordability, compactness, user-friendliness, as well as biodegradability, microfluidic paper-based analytical devices (μPADs) render suitable characteristics for point-of-care applications, especially in low-resource settings. There has been growing interest in the field since the conception of the first paper-based microfluidic device in 2007 by Martinez et al.^1^. The World Health Organization (WHO) has established the acronym ASSURED as a set of benchmarks for potential diagnostic devices, meaning: affordable, sensitive, specific, user-friendly, rapid and robust, equipment-free, and deliverable^2^. Therefore, an optimum fabrication approach should implement low-cost instrumentation as well as materials and procedures that yield rapid fabrication, ensuring scalability. Moreover, it is imperative to develop fabrication methods that allow for further increase in channel resolution, given the well-known benefits of miniaturization, such as the overall faster analysis time, high throughput, lower consumption of reagents, and therefore lower cost per sample, reduced waste product, portability, enhanced detection limits, among others^3–8^.

Numerous fabrication techniques have been investigated and developed, some allowing for considerably high resolutions. Martinez et al. were the first to develop a photolithography process for μPADs, achieving channel resolutions of 186 ± 13 μm^9^. Moreover, laser treatment for μPAD fabrication was introduced by Chitnis et al., in which a CO_2_ laser was used to modify the surface of the substrate, resulting in hydrophilic channels with resolutions as high as 62 ± 1 μm^10^. A pen plotter approach was developed by Amin et al., in which a desktop pen plotter and a high-resolution technical plotting pen were employed to develop resolutions of 150 ± 12 μm^11^. Other procedures have also been developed, such as plotting^12^, plasma treatment^13^, and stamping^14^. Nonetheless, these approaches require expensive initial investments in equipment as well as special training for high-skilled operators, and present low scalability. Methods employing wax in different configurations and inkjet printing have also been presented as alternatives^15–20^. However, most of the automated printing techniques require high initial investments in equipment, maintenance, and energy consumption.

Screen-printing stands out as a feasible approach for the fabrication of μPADs, given the simplicity of the procedure, being applied in a variety of configurations and featuring different hydrophobic agents such as wax, PDMS, polymethylmethacrylate (PMMA), vanishing paint, polystyrene, rubber latex, among others^21–29^, all yielding different ranges of channel resolutions. The overall results of the various techniques mentioned above are detailed in Table 1. Nevertheless, the resolution achieved using screen-printing, despite its potential for mass production, is still relatively far from other more elaborated fabrication methods.

**Table 1:** Variations of fabrication techniques developed for the manufacturing of μPADs.

Fabrication method	Hydrophobic agent	Achieved resolution	Paper type	References
Laser treatment	Silicone coating	62 ± 1 μm	Parchment paper	^10^
Pen plotting	Hydrophobic ink	150 ± 12 μm	Whatman No. 1	^11^
Photolithography	SU-8	186 ± 13 μm	Chromatography paper	^9^
Screen-printing	Rubber latex	256 ± 21 μm	Paper towel	^25^
		540 ± 70 μm	Whatman No. 4	
		780 ± 98.5 μm	Whatman No. 1	
	Varnishing paint	500 μm		^28^
	PDMS	650 μm		^26^
	Wax	650 ± 71 μm		^21^
	Polystyrene	671 ± 51 μm	Whatman No. 4	^29^
	PMMA	1000 μm		^27^
Inkjet printing	UV curable acrylate ink	272 ± 19 μm	Advantec No. 5C	^16^
Chemical treatment	Wax (μPAD submerged in NaIO4)	301 μm	Whatman No. 1	^19^
Flexography printing	Hydrophobic ink	500 μm		^20^
Inkjet etching	Polystyrene	550 μm	Advantec No. 2	^17^
Wax dipping	Wax	639 ± 7 μm	Whatman No. 1	^18^
3D pen drawing	Acrylic resin	2000 μm	Whatman No. 1	^30^
UV light curing	Water-based polyurethane acrylate	200 μm	Whatman No. 1	^31^

Previous reports present Whatman filter papers as the substrate of choice, given that the properties of these papers are well documented and characterized. Yet, these papers are relatively thick (180–210 μm), generating challenges in terms of fabrication, requiring the hydrophobic agent to be squeezed repeatedly for it to penetrate the substrate from top to bottom. Furthermore, the hydrophobic agent penetrates further parallel to the surface of the substrate in thinner papers, allowing for smaller dimensions for a given mesh screen geometry, increasing the opportunity for higher resolutions.

Consequently, our group proposes the use of a novel paper substrate in the fabrication of μPADs: Xuan paper, characterizing its potential through a direct comparison with Whatman filter papers (no. 1 and no. 4) in terms of wicking capability. The latter were chosen since the manufacturer catalogs them as featuring fast-wicking and medium-fast-wicking capabilities^32,33^. Xuan paper is a traditional Chinese handmade paper usually used for Chinese calligraphy and painting, being the preferred paper for the conservation of paper-based artifacts. It is primarily composed of bark fibers of blue sandalwood (Pteroceltis tatarinowii), growing in calcium-rich soil, and rice straw (Oryza Sativa) growing in silica-rich soil^34,35^. Luo et al. demonstrated that Xuan paper shows a similar temperature and light-induced degradation behavior as most western papers^36^. Zhou et al. verified that Xuan paper exhibits better cell compatibility compared with Whatman filter paper and lens paper^37^. Moreover, Xuan paper can be fabricated in a considerably wide range of thicknesses (50–150 μm), offering customizability. All the aforementioned advantages of Xuan paper are amplified considering that its price is only one percent of that of filter paper^36^. Therefore, the present work intends to extend on the findings previously mentioned in the literature, from a fabrication standpoint, identifying the factors that play a substantial role in the efficient manufacturing of μPADs using Xuan paper as a substrate.

As for hydrophobic agents, PDMS and wax have been proposed quite comprehensively for the fabrication of μPADs^21–24,26^, especially the latter, due to its affordability and availability. However, reports have indicated that μPADs fabricated using wax are inflexible and impaired under bending stress^25^. Moreover, extra reheating steps are required for wax to penetrate the substrate, hindering the efficiency of the fabrication process. On the other hand, PDMS also presents some disadvantages, as it involves a rather lengthy curing process, on top of its considerably higher price. Therefore, for this research, nail oil is proposed as an alternative hydrophobic agent.

The core constituents of basic nail oil are organic solvents such as ethyl acetate and butyl acetate as well and a film-forming agent, mainly, nitrocellulose. When applied, the solvent components evaporate and only the film-forming polymer remains^38^. The swift drying implies a more efficient fabrication since there is no need for curing after nail oil is applied onto the substrate. Foster et al.^39^ applied nail oil on paper-based screen-printed electrodes to insulate the connections. Satarpai et al.^40^ used nail oil as a hydrophobic agent for the first time, utilizing three different fabrication methods, achieving a channel resolution of 650 μm.

In this work, similar to the consideration of the thickness of the substrate, the temperature is also identified as a key factor in the pursuit of a higher resolution for screen-printed μPADs. The decrease of hydrophobic agent’s viscosity as its temperature increases, allows it to penetrate further parallel to the surface of the substrate, which would be usually regarded as something to avoid. We intend, by optimizing the temperature of the hydrophobic agent as well as the substrate’s thickness, to control the over-penetration of the hydrophobic agent to attain a higher resolution.

The performance of each combination of paper substrate-hydrophobic agent is quantified in terms of repeatability (namely, through relative standard deviation). Once the variables governing the process are properly optimized for the fabrication of well-defined high-resolution microfluidic channels, the wicking performance is analyzed. Lastly, to demonstrate the feasibility of applying our proposed fabrication procedure and the advantages it enables, a microfluidic pH indicator strip is successfully developed. For this device, sample volumes in the nano-liter scale are sufficient to obtain an accurate colorimetric measurement of a given sample pH, presenting solutions in settings where the use of costly and scarce samples needs to be kept to a minimum. Contamination of the sample is prevented, as a microfluidic channel is used to draw the sample into the sensing zone, avoiding direct contact between the sample reservoir and the pH indicator. It is proved in this report that screen-printed μPADs using Xuan paper, in combination with nail oil, PDMS, and wax as hydrophobic agents, yield high resolutions on par with those of more complicated fabrication processes. Furthermore, the findings of the present work serve as a set of guidelines on which researchers and developers in the field can rely, so that with the proper consideration and adjustment of the thickness of the paper substrate, as well as temperature set up of the hydrophobic agent, different results and customizations for a given device or application can be achieved.

## Experimental section

### Chemicals and materials

Whatman filter paper No. 4 (210 μm thickness, 25 μm pore size, model No. 1004–150) and Whatman filter paper No. 1 (180 μm thickness, 11 μm pore size, model No. 1001–110) were used as a frame of comparison. Xuan paper sheets, manufactured by Taiwan Cotton Paper Mill Co., Ltd, were employed in thicknesses ranging from 50 μm to 150 μm. As hydrophobic agents, PDMS (SYLGARD 184 Silicone Elastomer), standard paraffin wax, and generic nail oil (both manufactured by Yi Sheng Sing Enterprise Co., Ltd) were used. Moreover, given that the print resolution is inversely proportional to the pore size of the mesh screen, the minimum pore size attainable by local manufacturers was selected. An aluminum frame stencil, manufactured by Guger Industries Co., Ltd with a 420 nylon mesh screen and pore size of approximately 35 μm was employed for the fabrication of channels.

### Instrumentation

The channel width achieved and capillary-driven flow were monitored on video using a digital microscope (CCD camera) H800X/U500X ST2, (Neon Advanced Technology Co., Ltd). The horizontal 1-D motion was further analyzed by image processing on ImageJ software. The statistical analysis was performed on SPSS Statistics software (IBM).

### Fabrication technique

**Figure 1:**
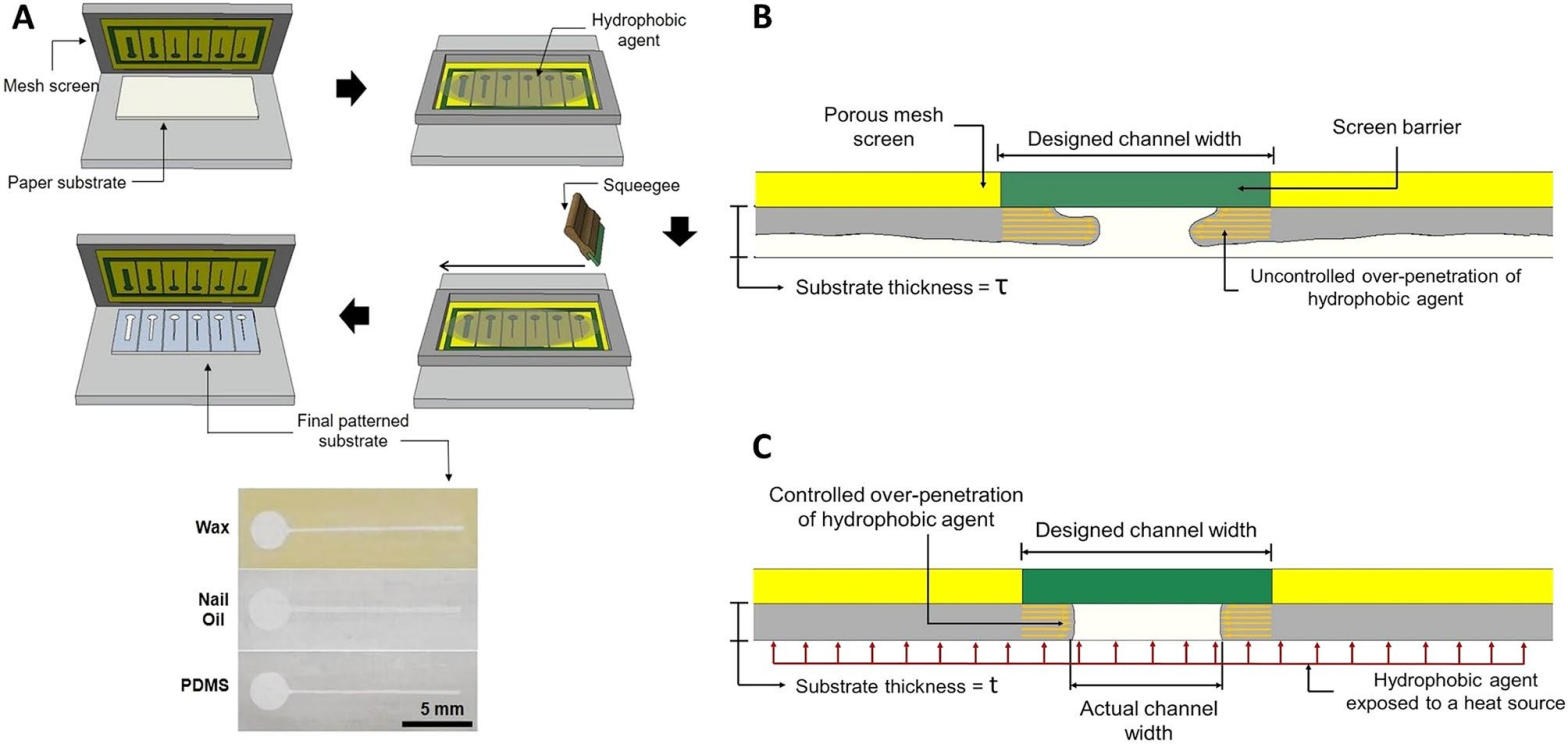
(**A**) Schematic representation of the screen-printing fabrication method used in the present study. (**B**) Cross-sectional view of screen-printing fabrication when the temperature of the hydrophobic agent and the substrate thickness are not properly optimized. (**C**) Cross-sectional view of screen-printing fabrication when the temperature of the hydrophobic agent and the substrate thickness are optimized. The over-penetration of the hydrophobic agent works in favor of reducing the channel width under controlled conditions.

The fabrication procedure is shown in Fig. 1a. The channel and hydrophobic-barrier patterns were designed using AutoCAD and manufactured on a woven mesh screen. For experimental purposes, patterns with channel widths varying from 5000 μm to 200 μm were designed. The hydrophobic agent was then applied over the screen and squeegeed, at a constant speed and one time only, through the woven mesh screen so that it would penetrate to the bottom of the paper, generating a hydrophobic barrier. The use of PDMS and nail oil was approached in the same fashion, in which the patterned substrate was immediately placed on a hot plate (Corning Inc.), for a 20-s incubation for a temperature setup higher than room temperature. However, the temperature set-up was adjusted according to the properties of each hydrophobic agent. PDMS has a higher viscosity than nail oil, thus the temperature was set slightly higher (20, 50, 80 °C), compared to nail oil (20, 40, 60 °C). For the case of wax, two hot plates were employed, the first one for heating solid wax and the second one to place the substrate. The temperature of the hot plate was set at 50, 55, and 60 °C. After melting, wax was poured onto the patterned woven mesh and rubbed through the screen into the paper matrix. It is hypothesized in this work, that a thicker paper substrate presents challenges in attaining higher resolutions, given that the hydrophobic agent, if only squeegeed one time, is not able to penetrate thoroughly across the substrate, as shown in Fig. 1b. However, if it is squeegeed further, the channel is prone to close fully. If a thinner paper is used, conversely, the hydrophobic agent can penetrate completely through the substrate by being squeegeed one time only, preventing any leakages of fluid across the channel. Adjustment of temperature can be further implemented to make use of the over-penetration of the hydrophobic agent in a controlled manner, as depicted in Fig. 1c, to attain higher resolutions.

To determine the channel width of the resulting pattern, firstly, a high-resolution picture of the channel was captured, so that sets of points were marked on five different locations along the length of the resulting channel. Subsequently, the transversal distance between these points was verified and then averaged to determine the channel width of the resulting channel. Then water was flown across the length of the channel and monitored on video by using a digital microscope (CCD camera). The displacement of the waterfront was then recorded, against time, for the study of the wicking properties within the fabricated channel. All the measurements and image processing were performed using ImageJ software, in which a known measurement scale is transformed into a scale of pixels, and then compared to the dimension to be measured.

### Preparation of microfluidic pH indicator strip and measurement

To prepare the pH indicator solution, 3 mL of bromothymol blue (Emperor Chemical Co. Ltd, Taiwan) were heated up at 60 °C for 6 min, to increase the saturation of particles of the pH indicator in the solution and attain a more uniform color distribution of the pH dye solution on a paper substrate. The solution was then used to coat approximately half of a 15 × 30 mm (width × length) Xuan paper (150 μm thickness) strip and left to dry. A channel of around 300 μm was then screen-printed on the coated strip using nail oil as a hydrophobic agent. Samples with different pH values, ranging from 5 to 9 were prepared by adjusting their pH with sodium hydroxide (NaOH) and hydrochloric acid (HCl). The pH was monitored with a pH meter (Laqua F-71, HORIBA, Japan). Each sample was then flown through the uncoated part of the microchannel and drawn into the sensing zone, monitoring the color change through video. Images of each measurement were taken using a Samsung Galaxy Note 10 camera, with built-in flash enabled, ISO set to 200, using the autofocus mode, under constant lighting conditions, after 2 min of drawing the sample into the channel. Further image processing was carried out in ImageJ software to determine the RGB values of the resultant color of each measurement. The obtained RGB values were transformed into grayscale intensity values through the weighted method, also called the luminosity method, in which each channel contribution is weighted according to its wavelength^35^. Moreover, to reduce any variations due to lighting conditions, RGB values from the background (zone of the pH strip that did not react with the sample) were transformed to grayscale intensity values in the same fashion, and then used to normalize the grayscale intensity values corresponding to each pH measurement.

## Results and discussion

### Determination of wicking capabilities of Xuan paper: preliminary validation of material selection

**Figure 2:**
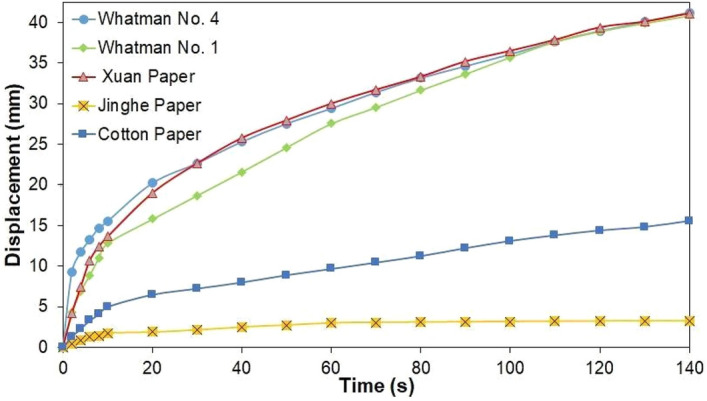
Comparative wicking performance for different paper substrates.

To characterize the ability of Xuan paper as a potential substrate for μPAD applications, a comparison was established against different paper substrates commonly used in the field, namely Whatman filter paper No. 1 and Whatman filter paper No. 4. Moreover, calligraphy papers such as cotton and Jinghe papers were also added to the comparison, to identify the type of paper that better approached or surpassed the benchmark established by the filter papers. A channel of 5 mm was fabricated using PDMS as a hydrophobic agent on all different types of paper substrates. Water was flown through the fabricated channel in both paper substrates and the displacement covered by the waterfront was measured against time, as depicted in Fig. 2. From the comparative, it was found that Xuan paper delivered similar results in contrast with Whatman filter paper No. 4, to the extent of surpassing Whatman filter paper No. 1 in terms of its wicking speed, proving that for a given application, Xuan paper could be considered as a potential substrate for the fabrication of μPADs in terms of its wicking characteristics. This finding can be mainly attributed to the fact that Xuan paper and Whatman filter paper No. 4 have similar hydrophilic features, characterized by their contact angle with water, as shown in Table 2, where Xuan paper and Whatman filter paper No. 4 have a contact angle of approximately 46.04° and 45.43°, respectively. It is worth clarifying that contact angles were measured using the Wilhelmy plate method^41^ and that the main purpose of this measurement was to shed light on the trends of wetting characteristics of the paper substrates, relative to each other, and not necessarily to determine the actual value of the contact angles.

**Table 2:** Comparison of characteristics of substrates used in the present study.

Substrate	Thickness [μm]	Contact angle	Cost (USD/m^2^)	Displacement of waterfront [mm]: Time intervals (5 mm channel width)			Components
				20 s	60 s	120 s	
Whatman No. 4 (pore size: 20 μm)^42^	210	46.04°	≈24.80	20.2	29.4	38.9	Cellulose fiber of treated cotton linter, high on alpha-cellulose^44^
Whatman No. 1 (pore size: 11 μm)^42^	180	50.4°	≈ 24.30	15.8	27.5	38.8	Cellulose fiber of treated cotton linter, high on alpha-cellulose^44^
Xuan paper (pore size: 14 μm)^43^	150	45.43^°^	≈ 0.26	18.9	29.9	39.3	Bark fibers of blue sandalwood and rice straw^36^
Cotton paper	100	51.73°	≈ 0.47	6.5	9.6	14.4	Cotton linter fibers^45^
Jinghe paper	130	54.48°	≈ 0.6	1.9	2.9	3.2	Bark fibers of mulberry plants, evodia tree^45^

**Figure 3:**
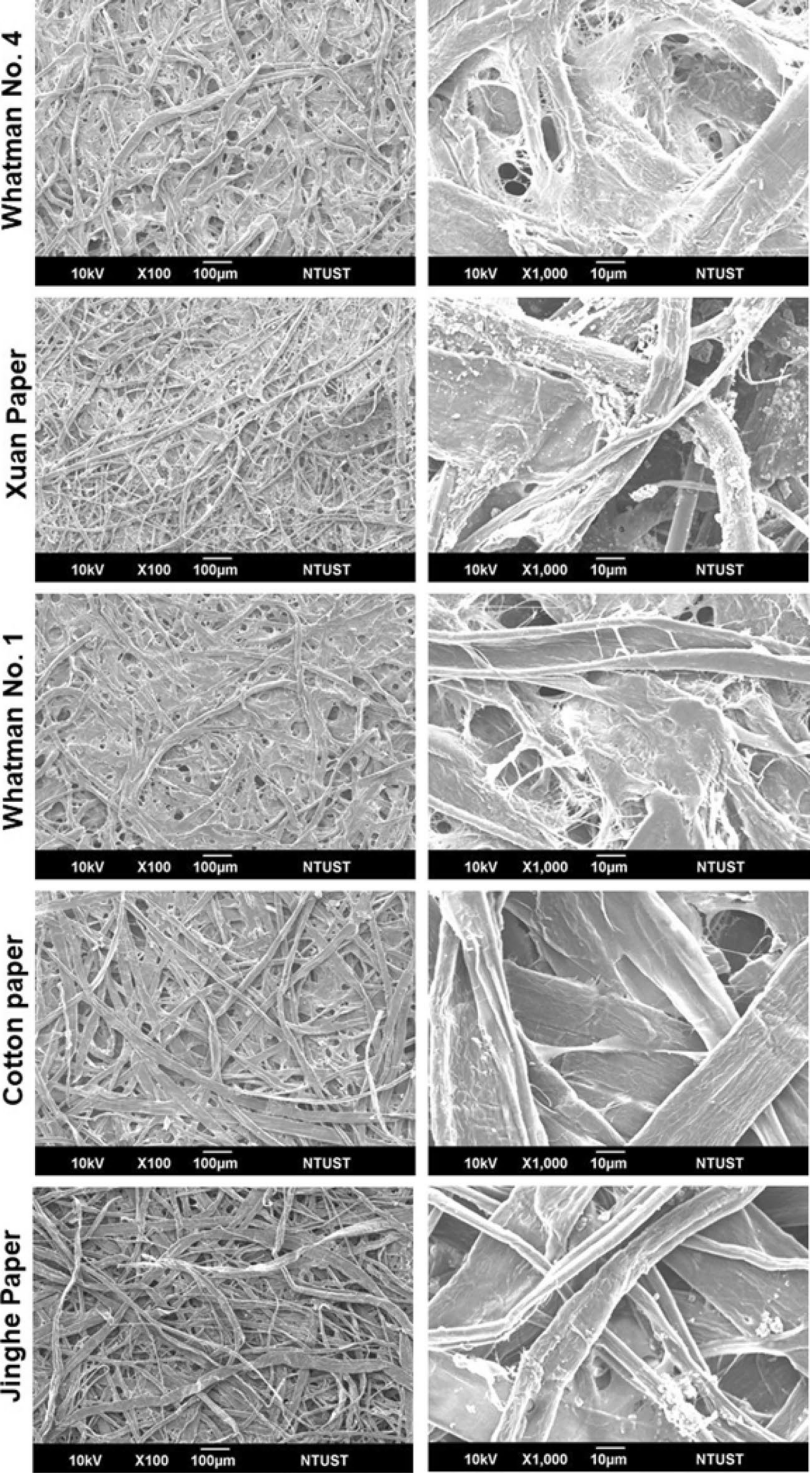
SEM imaging of substrate surface morphology for each paper substrate at × 100, and × 1000 magnification, respectively.

The morphology of the surface for the substrates mentioned above was studied using scanning electron microscope (SEM) imaging, to further understand the difference of wicking behavior for each substrate, as depicted in Fig. 3. It can be observed that those substrates with superior wicking capabilities presented a higher density of fibers intertwined with one another, facilitating the permeability of the fluid through the substrate, as was the case for Whatman No.4 paper, Xuan paper, and Whatman No.1 paper. Moreover, these three substrates shared a rather similar fiber size distribution, compared to the other two substrates, Jinghe paper, and cotton paper.

**Figure 4:**
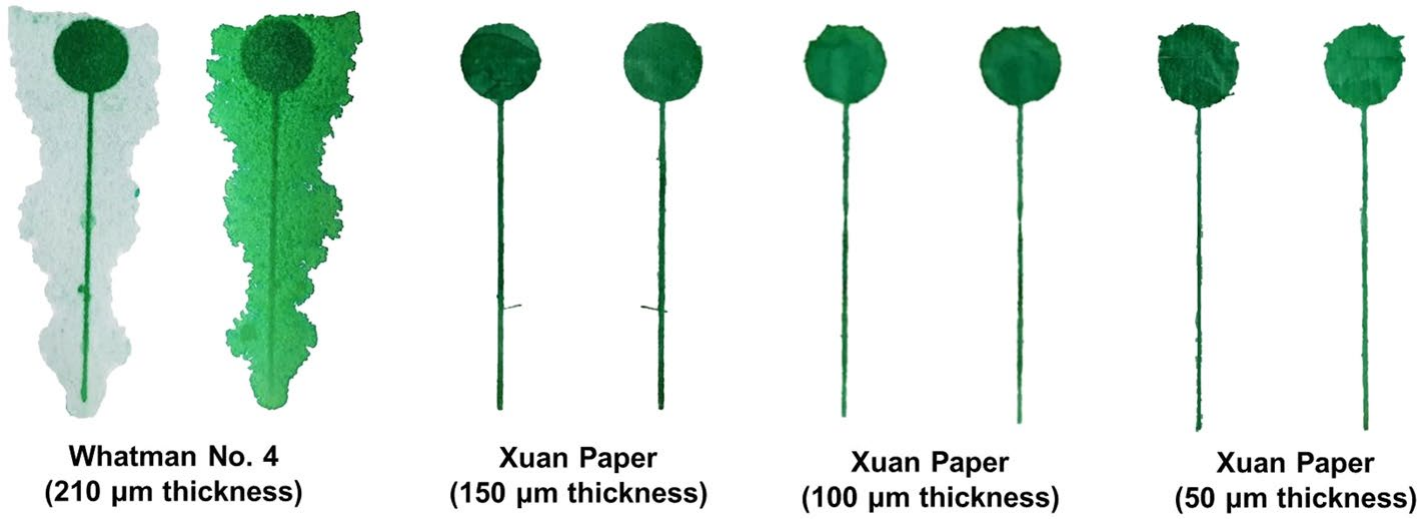
View of front and back face (shown on the left and right side of each pair of channels, respectively) of the paper substrates after being patterned. Colored-ink water was flown through the channels, and the background of the images was removed through image processing for clearer visualization. For the case of Xuan paper, all the patterns were found to be the same on the front and back face of the substrate, after the hydrophobic agent was squeegeed one time, implying that the barrier penetrated completely through the thickness of the substrate. Whatman filter paper, however, presented considerable leakage of the flow on the back face of the substrate, indicating that one squeeze of the hydrophobic agent is not enough to build an effective barrier.

Furthermore, it was also postulated that the thickness of the substrate would play a major role in the efficiency of the fabrication process. Microfluidic channels were patterned on Whatman filter paper No. 4, as well as on Xuan paper having different thicknesses (namely, 50 μm, 100 μm and 150 μm). The hydrophobic agent was squeegeed through the substrate only one time and at a constant speed. Colored-ink water was subsequently flown through the channels, to see if any leakage of the fluid took place. As shown in Fig. 4, Xuan paper, for all three thicknesses used in this study, showed the same pattern on both its front and back face, implying that the hydrophobic barrier was effective across the thickness of the substrate. For the case of Whatman filter paper, conversely, there was considerable leakage of the fluid outside of the limits of the patterned channel. Given that Whatman filter paper is rather thick (210 μm), the hydrophobic agent is not able to penetrate completely through the thickness of the substrate when it is squeegeed in only one movement. If the hydrophobic agent is squeegeed further, the patterned channel tends to close completely, hindering the possibility of obtaining higher resolutions employing screen-printing. This finding suggests that Xuan paper, besides offering different choices of thickness, also presents the opportunity to increase the resolution of the channel obtained, by only needing one squeeze of the hydrophobic agent, as well as improving the efficiency of the fabrication process, saving material used with each pattern.

### Comparison of substrate/hydrophobic agent performance on patterned channel width

Once it was proved that Xuan paper has adequate wicking capabilities for its use as a substrate in the fabrication of μPADs, it was proceeded to understand the interaction of the hydrophobic agents (PDMS, wax, and nail oil) in combination with Xuan paper. Microfluidic channels were patterned on Xuan paper substrates having three different thicknesses, 50 μm, 100 μm, and 150 μm, in conjunction with the variation of temperature.

**Figure 5:**
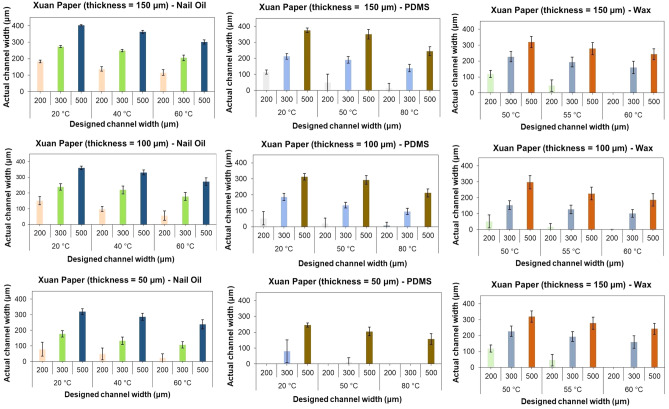
Comparison of paper substrate-hydrophobic agent performance using screen-sprinting fabrication in different combinations of variables (temperature, substrate thickness, and designed channel width). The data presented on each bar in the graph corresponds to the average (n = 6) of the actual channel width given a combination of variables.

Special importance was given to the narrower range of widths for the microfluidic channels (500, 300, and 200 μm), exploring the possibility of increasing the resolution of the fabrication process achieved so far in the field. Due to the dissimilar properties of the hydrophobic agent studied in this research,a different set of temperatures were used for each one of the agents, with wax requiring pre-heating to be able to flow completely into the substrate. Figure 5 portrays an overall view of the performance of each paper substrate—hydrophobic agent combination. The actual channel width achieved was found to be always narrower than the designed channel width for all three hydrophobic agents, mainly attributable to the unavoidable over-penetration of the hydrophobic agent into the substrate matrix. However, the results of using nail oil as a hydrophobic agent showed a closer relationship between the actual and designed width for all three different paper substrates, especially on the smallest designed channel widths. For this study, the reference for a successful result was regarded as a fabricated microchannel through which water was able to flow. Channel widths as narrow as 42 μm were achieved through screen-printing. However, water was not able to flow within channels narrower than approximately 200 μm.

### Influence of temperature and substrate thickness on the fabrication outcome

**Figure 6:**
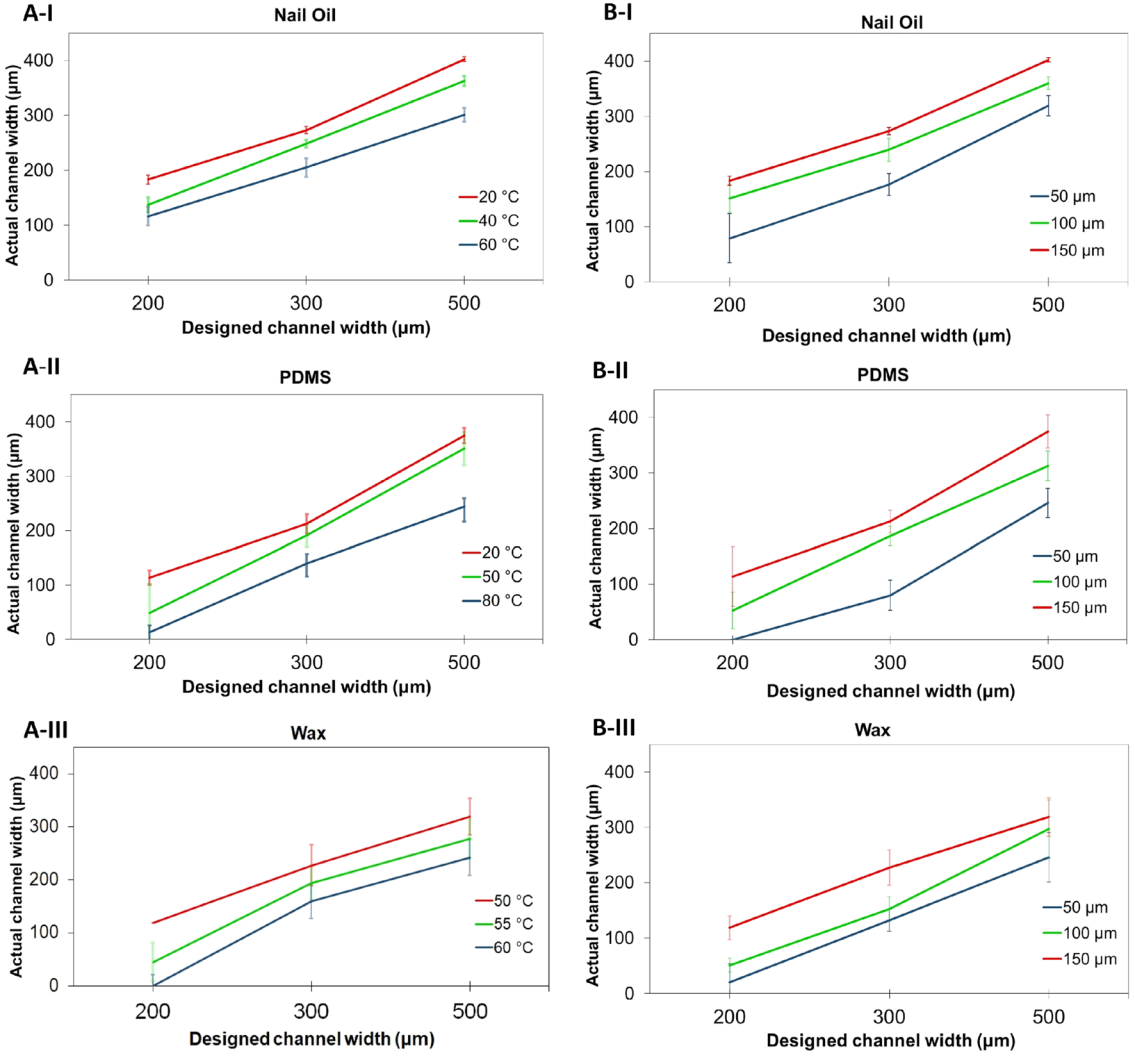
(**A**) Tendency for actual channel width achieved as the temperature setup for hydrophobic agent varies and the thickness remains fixed to 150 μm for nail oil (**I**), PDMS (**II**), and wax (**III**). (**B**) The tendency for actual channel width achieved as substrate thickness varies and the temperature remains fixed at the lower setting for nail oil (**I**), PDMS (**II**), and wax (**III**).

As was expected, temperature plays a major role in the overall performance of the screen-printing fabrication process. An increase in the temperature, and the consequent decrease in viscosity of the hydrophobic agent results in an even narrower channel width, compared to the designed channel width, with the flow wicking with more ease through the substrate matrix, as depicted in Fig. 6a. It is important to point out that, for a given temperature, it was noticed that PDMS requires a considerably longer time to be cured within the substrate matrix, compared to nail oil, where even at room temperature, it renders a device ready to use almost immediately after it is squeezed through the substrate. For wax, the fact that an additional pre-heating step is required for it to be able to penetrate entirely through the substrate matrix hinders the efficiency of the overall procedure.

The degree of influence the paper substrate thickness has on the overall system, in combination with the hydrophobic agent used, was also explored in this investigation, depicted in Fig. 6b, respectively. It was found that for different substrate thicknesses, there exists a similar behavior as the one exhibited by the variation of temperature, in which there is a trade-off between repeatability and achieving a narrower channel width. As the thickness of the substrate increases, so does the agreement between actual and designed channel width, with an increase in repeatability across all combinations of variables. However, for a given designed channel width, a smaller dimension is achieved by decreasing the thickness of the substrate, giving up control of the achieved dimensions at the expense of fabricating a narrower channel.

### Characterization of repeatability of screen-printing fabrication based on hydrophobic agent

**Figure 7:**
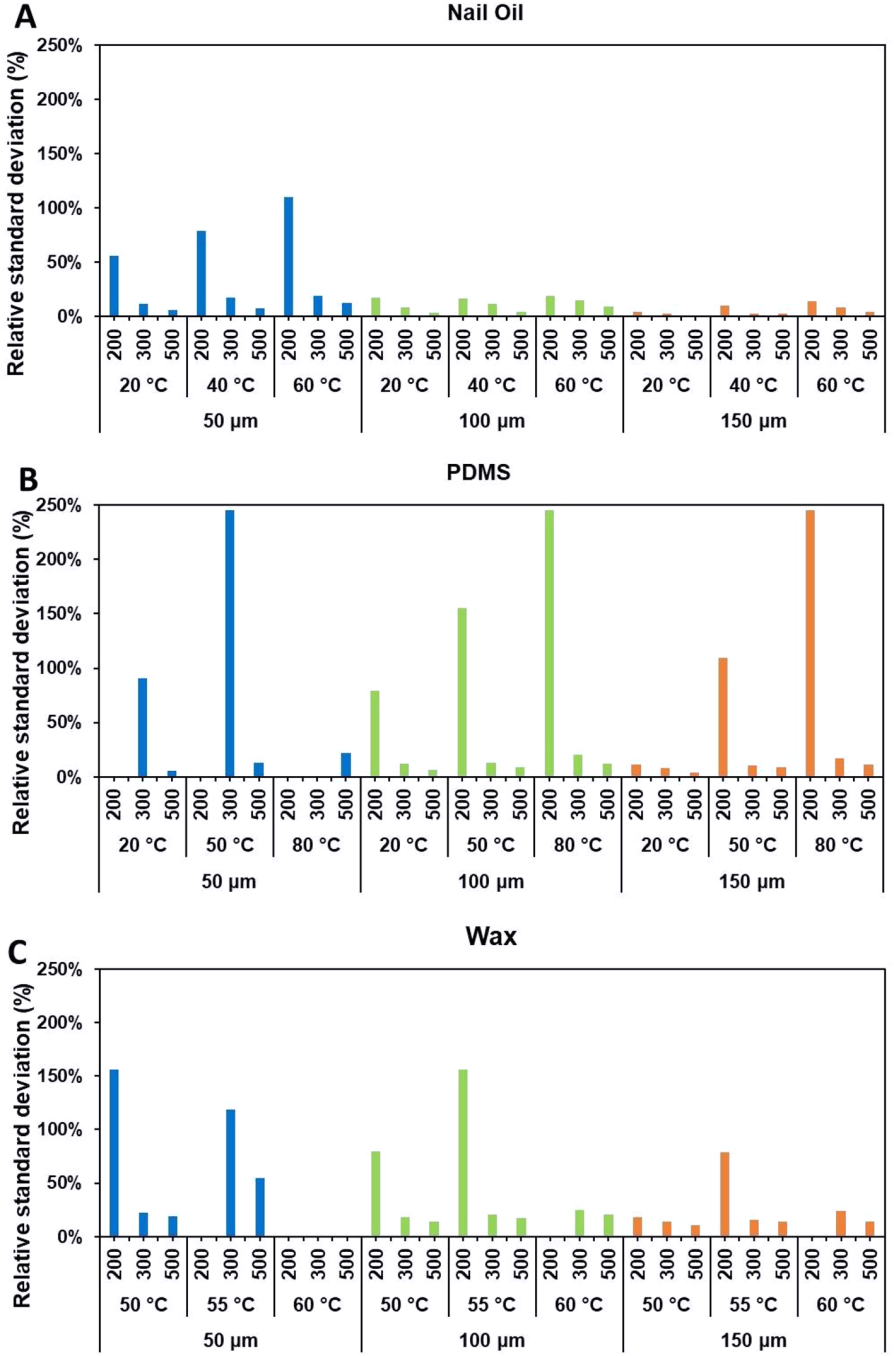
Repeatability of the fabrication process for each combination of variables: substrate thickness (50 μm, 100 μm and 150 μm), temperature set-up, designed channel width (200 μm, 300 μm and 500 μm) for nail oil (**A**), PDMS (**B**) and wax (**C**), respectively.

Microfluidic channels were patterned six times for each combination of variables, accounting for a data size of 162 channels for each hydrophobic agent. Subsequently, analysis of variance test (alpha level of 0.05), was performed on the data, obtaining average, standard deviation, and relative standard deviation (%RSD) for each set of experiments, as well as for each hydrophobic agent in its entirety. The descriptive statistics provided by the test were found to be significant for all variables (temperature, thickness, and designed channel width) considered in this study, at a *p*-value < 0.001. The relative standard deviation corresponding to each combination of variables for all three hydrophobic agents was used to quantify the repeatability of the screen-printing process, as depicted in Fig. 7. It was found that the %RSD for nail oil was considerably lower throughout in each case, whereby for a combination of 150 μm substrate thickness, at 20 °C and designed channel width of 500 μm, the %RDS was as low as 1%. As for PDMS, the lowest %RSD achieved was 3.82% and 10.85% for wax. It is worth clarifying, that the conditions in which PDMS and wax yielded %RSD of zero, were those in which the fabricated channel closed up in its entirety for all the attempts. Based on these findings, it is apparent that nail oil is able to deliver suitable results in terms of repeatability for the fabrication of μPADs.

### Influence of hydrophobic agents on wicking properties of fabricated μPAD

**Figure 8:**
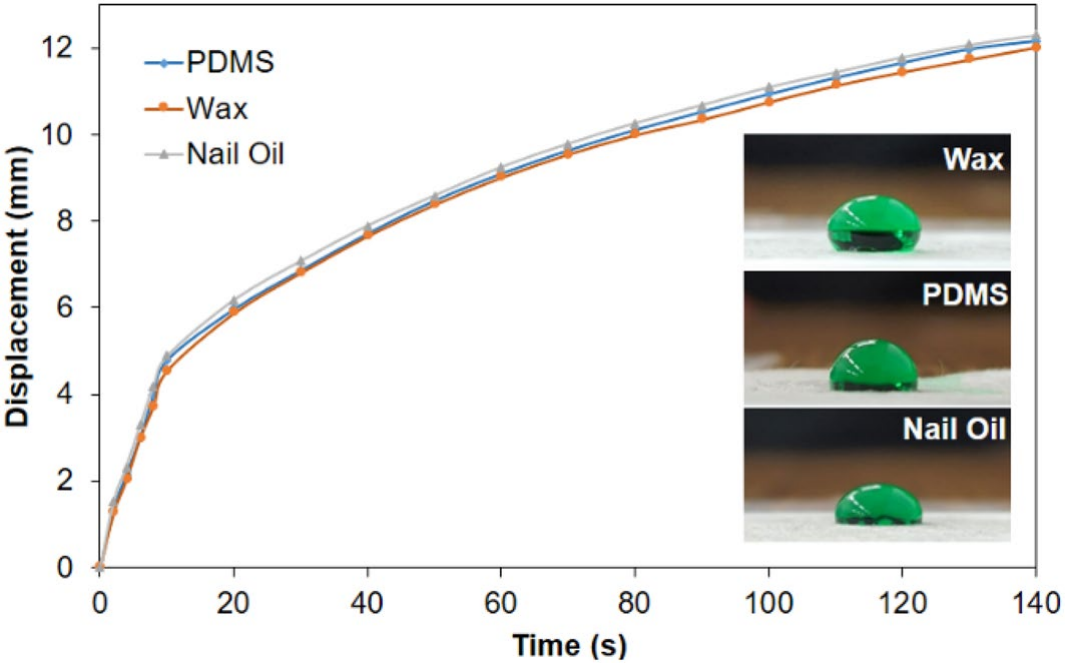
Comparison of wicking performance based on hydrophobic agent in a channel of 200 μm. The contact angle for the hydrophobic barrier using nail oil, PDMS, and wax is shown respectively.

Once the fabrication process was characterized and properly optimized, the focus shifted to the study of the wicking capabilities of the μPADs fabricated by screen-printing. Microfluidic channels of width ranging from 5 to 0.2 mm were fabricated using all three hydrophobic agents analyzed in this work. The flow of water within the channels was analyzed and compared to one another to determine the effect of the hydrophobic agent in the behavior of the flow. It was observed that for any given channel width, the flow was practically unaffected by the variation of the hydrophobic channel, as depicted in Fig. 8, where the displacement of the waterfront had virtually the same tendency using either wax, PDMS, or nail oil to build the hydrophobic barrier. Contact angles were measured, with the aid of ImageJ software, for all three hydrophobic agents, namely: 104.8°, 99.2°, and 97.8° for wax, PDMS and nail oil, respectively; from which it can be inferred that the degree of hydrophobicity is very close between one another. . Moreover, each hydrophobic barrier was exposed to hydrochloric acid, sodium hydroxide, and Span® 80 solutions, to evaluate the resistance of these materials when combined with a strong acid, strong base, and surfactant solutions, respectively shown on Table 3. All three hydrophobic barriers showed no signs of damage nor leakage from any of the solutions when a drop of each solution was placed on the surface of the barrier and monitored for one hour after exposure.

**Table 3:** Resistance of hydrophobic barriers to acidic, basic, and surfactant solutions.

	Hydrochloric acid (35% w/w)	Sodium hydroxide (35% w/w)	Span 80 (1% w/w)
PDMS	✓	✓	✓
Wax	✓	✓	✓
Nail Oil	✓	✓	✓

### Influence of resulting channel width on wicking properties of fabricated μPAD

**Figure 9:**
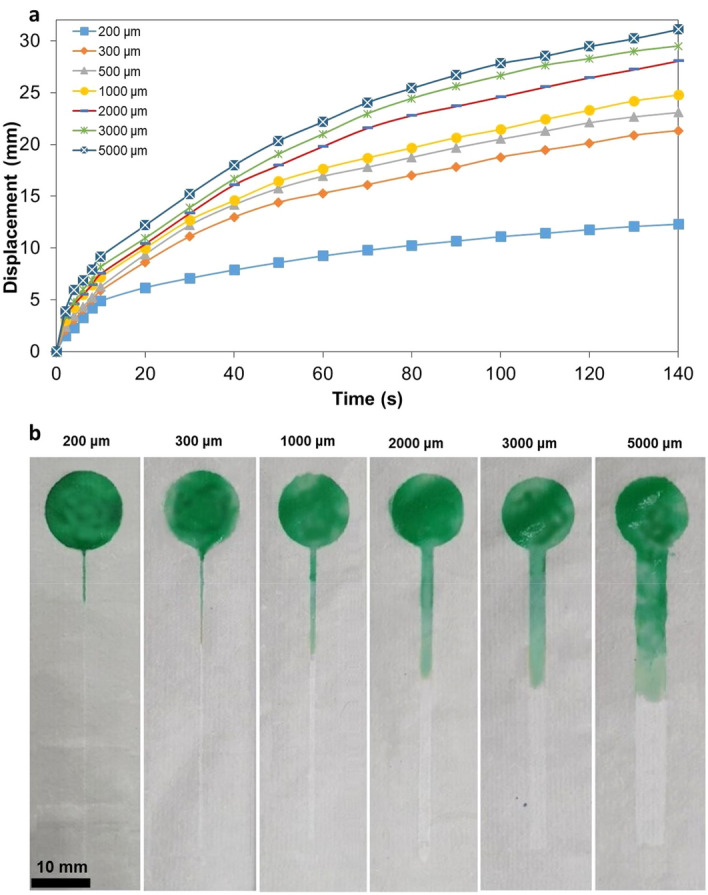
(**a**) Measured flow displacement of waterfront within microfluidic channels of different widths, using a substrate with a thickness of 150 μm and nail oil as the hydrophobic agent. (**b**) Actual depiction of flow within fabricated channels after 80 s of loading the channel. Water was ink-colored for visualization purposes.

Furthermore, a relation between the microfluidic channel width and the behavior of the capillary flow was also drawn from the aforementioned process. The tendency of the flow was rather similar for all different channel widths. Initially, the waterfront is driven quite fast by the capillary force, yet as the displacement within the microfluidic channel increases, the drag within the channel increases, leading to a deceleration of the waterfront. However, it is worth noting that the degree of resistance to flow is also increased as the microfluidic channel width decreases, as shown in Fig. 9. Water was able to flow for channel widths as low as approximately 200 μm; for smaller dimensions, the flow was stopped almost immediately after being drawn into the channel.

### Determination of pH using sample volumes in the nanoscale

**Figure 10:**
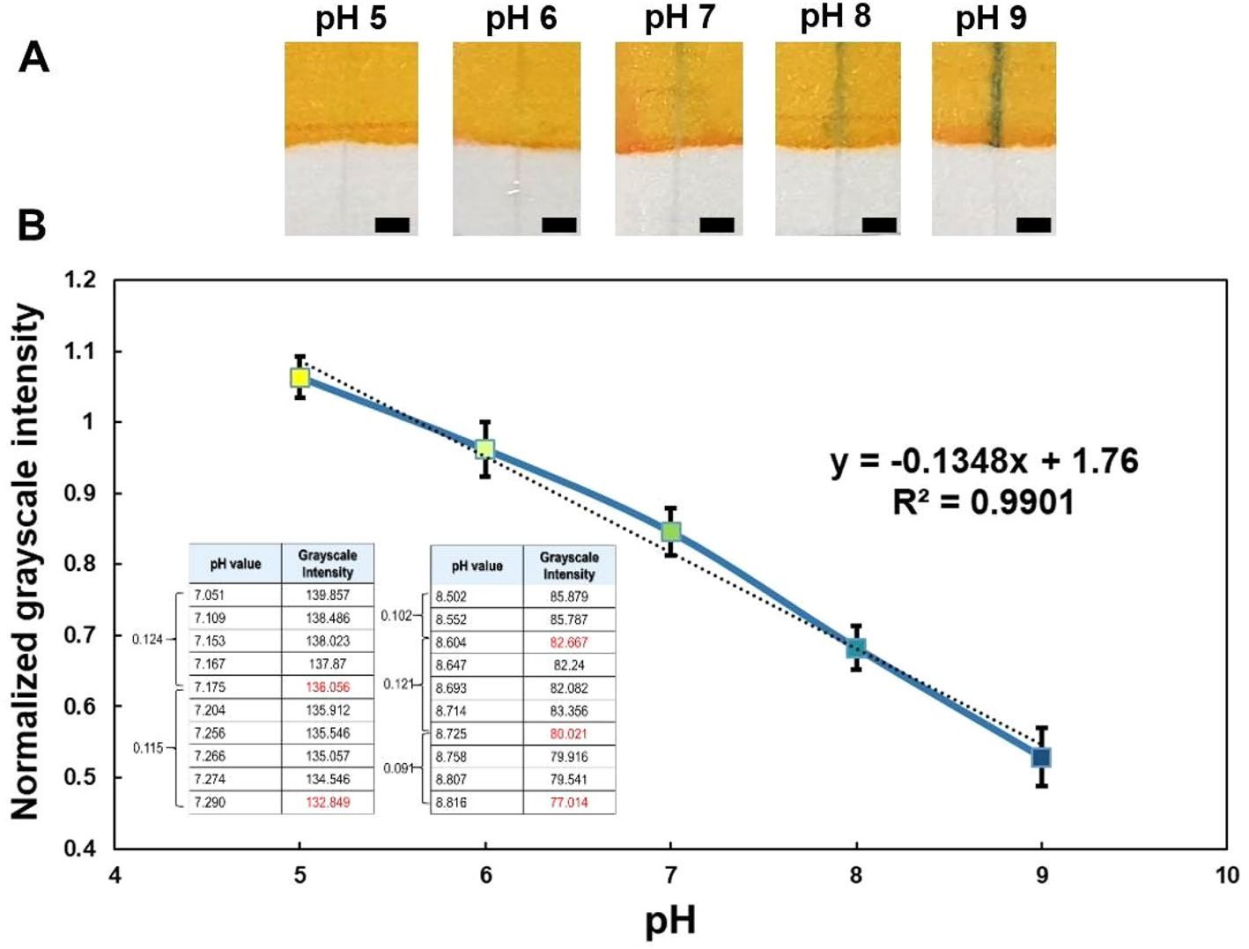
(**a**) pH testing using bromothymol blue as pH indicator and screen-printed channels of approximately 300 μm width (scale bar: 2 mm). (**b**) Determination of calibration curve based on normalized grayscale intensity corresponding to pH values ranging from 5 to 5. Each point represents the average grayscale intensity with n = 4. The tables display the corresponding data used to estimate the measurement resolution of the device.

One of the main advantages of the miniaturization of microfluidic channels is the substantial decrease of required volume to perform different types of analyses. The benefits from this feature are amplified when there is a need to determine certain properties of a given analyte before its use in an assay, but there is no room for waste given the limited quantities available of the analyte in question. Therefore, we demonstrate the advantages that our proposed fabrication technique enables and the solutions that can be provided, by developing a smart microfluidic pH indicator strip, which requires on average, 0.83 μL of sample volume to yield an accurate reading of its corresponding pH value. Moreover, given that a microchannel that has not been coated with the pH indicator (Fig. 10a) is used to draw the sample into the sensing zone, contamination of the given sample is avoided, since no direct contact between the sample reservoir and the pH indicator is required to accomplish the measurement, as it would happen with the use of conventional pH strips.

Once the samples with different known pH values, ranging from 5 to 9, were spotted on the patterned channel, calibration of the device was attained by taking high-resolution images of the reaction between the sample and the pH indicator. The images were then processed according to the procedure described in the experimental section, yielding the calibration curve shown in Fig. 10b. The pH indicator used in this demonstration, Bromothymol blue, presents blue colors for pH above 7, and turns yellow for pH below 7; for neutral values, the color of the indicator is green, which all agree with the color scale attained on the patterned microfluidic channel.

Subsequently, buffer solutions were prepared in intervals of 0.01 units of pH, in a range of pH values from 7.05–7.30 and 8.5–8.80 to determine the measuring resolution of the pH strip. It was found that to generate a change in the grayscale intensity reading, the smallest change of pH detectable was approximately 0.11 units of pH. To validate the reliability of the pH strip and the calibration curve developed in this study, the pH of DI water samples, as well as tap water was measured using both a pH meter and the pH strip proposed in this work. From Table 4, it can be seen that measurements acquired from the pH strip were within a slight margin of error compared to those drawn from the pH meter. For the case of DI water, the average error after four different measurements of pH was only 0.086. For tap water, despite presenting a higher average error, given the presence of impurities in tap water that can act as sources of noise, the result was nonetheless fairly acceptable for measurements that do not require extensively high levels of accuracy considering the gap in development cost for a pH meter and the pH strip presented. In this regard, the accuracy of the device is verified by considering the measurement of both samples, where the combined average error in reading from the pH strip is 0.264 units of pH. Additionally, the precision of the pH strip was quantified in terms of the combined standard deviation of the validation data. After combining the standard deviation obtained from the measurements of both samples, the precision of the device is 0.269 units of pH. Moreover, the reproducibility for measurements using the pH strip was within quite an acceptable range, where the relative standard deviation was not larger than approximately 3%.

**Table 4:** Measurement of pH for real samples using a pH a meter and the pH strip proposed in this study.

	pH meter	pH strip				Average error	Standard deviation	%RSD (%)
		1st try	2nd try	3rd try	4th try			
DI water	6.270 ± 0.030	6.118	6.212	6.118	6.572	0.086	0.196	3.1
Tap water	7.327 ± 0.040	8.088	7.575	7.665	7.746	0.442	0.224	2.9

## Conclusion

For the first time, Xuan paper was successfully adapted as a novel substrate in conjunction with nail oil, PDMS, and wax, for a process requiring neither sophisticated equipment nor high-skilled personnel, rendering a rapid, simple, and inexpensive screen-printing μPAD fabrication, with well-defined hydrophobic barriers. This new paper substrate offers capabilities on par with the more renowned Whatman filter paper, with a substantial cost reduction. Moreover, the variety of thicknesses in which Xuan paper can be acquired provides flexibility when it comes to the design for μPAD applications. PDMS and wax were successfully implemented as hydrophobic agents in combination with Xuan paper. However, nail oil proved to be worth considering, adding a high degree of repeatability. More importantly, with the appropriate control of temperature set-up and thickness of the substrate, the over-penetration of the hydrophobic agents is used to our favor, attaining a considerable improvement in terms of the achieved channel resolution of the process, decreasing the microfluidic channel width for fully functional, repeatable μPADs down to approximately 97.83 ± 16.34 μm (Nail oil at 40 °C on Xuan paper with a thickness of 100 μm) with 16.70% RSD. This finding accounts for a substantial improvement in the resolution of the process, compared to prior arts. Moreover, with the demonstration of pH measurement, the foundations are established for the further development of miniaturized μPADs that allow us to operate with volumes in the nano-scale, providing valuable alternative solutions in low-resource settings. Based on our successful attempts on improving fabrication resolution for screen-printed μPADs with the employment of newly proposed materials, we believe our demonstrated technology paves the way for further growth within the field of paper microfluidics and its adoptions in wider applications requiring minimal sample consumption.

## Data Availability

The datasets used and/or analyzed for the present work are available upon request.
